# Worse Sleep Quality Aggravates the Motor and Non-Motor Symptoms in Parkinson's Disease

**DOI:** 10.3389/fnagi.2022.887094

**Published:** 2022-06-10

**Authors:** Qu Yi, Chen Yu-Peng, Li Jiang-Ting, Li Jing-Yi, Qin Qi-Xiong, Wang Dan-Lei, Zhao Jing-Wei, Mao Zhi-Juan, Xiong Yong-Jie, Min Zhe, Xue Zheng

**Affiliations:** Department of Neurology, Tongji Hospital, Tongji Medical College, Huazhong University of Science and Technology, Wuhan, China

**Keywords:** Parkinson's disease, sleep characteristics, motor symptoms, non-motor symptoms, Pittsburgh Sleep Quality Index

## Abstract

**Backgrounds:**

Sleep disorders are the most common and disabling symptoms in patients with Parkinson's disease (PD). Understanding the associations between sleep characteristics and motor and non-motor symptoms (NMSs) in PD can provide evidence to guide therapeutic interventions and nursing strategies. We aimed to investigate the association between sleep characteristics and motor function and NMSs in PD using multiple approaches.

**Methods:**

A total of 328 participants were included, and all participants underwent Pittsburgh Sleep Quality Index (PSQI) evaluation and clinical assessments of PD symptoms. We conducted Spearman's correlation to evaluate the associations between sleep and PD symptoms, nonlinear regression to assess the relationships between sleep habits and PD, and mediated analyses to test the effects of NMSs on global PSQI and PD severity, quality of life, and motor symptoms.

**Results:**

Poor sleep was associated with more severe PD symptoms. In addition, the reflection point for bedtime was around 21:52, associated with motor symptoms, and insufficient and excessive total time spent in bed and nocturnal sleep duration were correlated with higher NMS burdens. The optimal points were 8–9.2 and 6.2–6.9 h, respectively. It was also discovered that NMSs played the mediating roles in global sleep with the quality of life, PD stages, and motor symptoms to a varying range of 6.8–95.4%.

**Conclusions:**

Sleep disorders have a significant effect on the burden of PD symptoms. The current findings provide new insights into the monitoring and management of sleep and PD and need to be further explored in the future studies.

## Introduction

Parkinson's disease (PD), the second most common neurodegenerative disease in the elderly, is brought into focus since it cannot be cured; therefore, an increasing number of studies pay close attention to early diagnosis of PD (Stefani and Högl, 2020). The main motor manifestations of PD are resting tremor, bradykinesia, muscle rigidity, and walking and gesture abnormalities. The appearance of non-motor symptoms (NMSs) has been proven earlier than that of motor symptoms. NMSs are the most disabling symptoms of PD and are often neglected. Therefore, it is of great value to explore the clinical characteristics of NMSs for early diagnosis and intervention (Sveinbjornsdottir, 2016). Sleep disorders are among the most frequent NMSs, with a prevalence of 20–90% (Cai et al., 2019), and are also regarded as life-changing symptoms due to their significant impact on the quality of life (Jasti et al., 2018). It needs to be identified and managed early to improve motor symptoms.

Sleep–wake disturbances have been reported to precede the diagnosis of Parkinsonism in prodromal PD (Al-Qassabi et al., 2017). The pathophysiology is unknown and may be caused by multiple factors, including the effects of motor function and NMSs on sleep. PD-related disturbed sleep is largely attributed to central sleep regulation center degeneration in the thalamus and brainstem, including difficulty falling asleep, decreasing total sleep duration and efficacy, frequent awakenings, and increasing daytime sleepiness (Zhu et al., 2016). Furthermore, dopaminergic therapies can precipitate sleep disturbances (Breen et al., 2014), and changes in non-dopaminergic neurotransmitter levels due to PD progression affect sleep (Diederich et al., 2005). According to the previous studies, sleep maintenance insomnia due to disrupted sleep occurs most commonly, followed by early morning awakening (Ylikoski et al., 2015). Studies using polysomnography have also revealed that patients with PD have shorter total sleep time and lower sleep efficiency using polysomnography (Yong et al., 2011; Selvaraj and Keshavamurthy, 2016). Moreover, worsening of sleep disturbances and other neuropsychiatric complaints may contribute to the progression of NMSs, and a broad range of sleep disorders are seen, with potential interactions existing in various disorders (Bargiotas et al., 2016). For instance, sleep disorders are related to emotional and cognitive impairment, as well as decreased quality of life, which augments the morbidity and mortality in the PD population (Pandey et al., 2016; Bohnen and Hu, 2019). Sleep quality is defined in terms of subjective complaints, and self-reported sleep measures are quick, brief, and cost-effective ways to measure sleep disturbances (Zea-Sevilla and Martínez-Martín, 2014). A host of scales has been applied for the evaluation of sleep disorders; however, only a few scales can be used to assess sleep quality in patients with PD (Högl et al., 2010). The Pittsburgh Sleep Quality Index (PSQI) is the most widely used scale in clinical practice and allows the rate of sleep quality to select appropriately targeted therapies (Videnovic et al., 2017).

Although studies have investigated the clinical characteristics of sleep disturbances among patients with PD, this relationship remains unclear and warrants further investigation. In addition, whether PD clinical characteristics, such as disease severity, prescription of dopaminergic medication, and other NMSs, are associated with sleep disorders remains controversial. Therefore, this study aimed to investigate the association between sleep characteristics and motor and non-motor dysfunctions of PD in this single-center observational cross-sectional study. An improved understanding of the common causes of sleep and PD characteristics is crucial and can provide evidence to guide therapeutic interventions and nursing strategies for patients with PD.

## Methods

### Study Participants

This observational, single-center, cross-sectional study enrolled 551 participants who visited the Department of Neurology, Tongji Hospital, Tongji Medical College, Huazhong University of Science and Technology from October 2014 to October 2021. PD was diagnosed based on the most recent clinical criteria (Postuma et al., 2015). Participants were excluded if they (1) were diagnosed with Parkinsonism-plus syndrome, including multiple system atrophy, progressive supranuclear palsy, and Lewy body dementia; (2) had a history of surgery, including stereotactic nerve nuclei lesions and deep brain stimulation; and (3) had a history of psychiatric symptoms, cancer, or any serious cardiovascular complications. This study was approved by the Medical Ethics Committee of Tongji Hospital, Tongji College of Medicine, Huazhong University of Science and Technology (Wuhan, China). Finally, 328 patients with PD were included, whereas 96 participants were excluded due to the above diagnosis and diseases, as well as 127 for incomplete data. Written informed consent was obtained from the participants or their legally acceptable representatives.

### Sleep Characteristics Assessment Headings

Sleep characteristics were measured using the PSQI (Högl et al., 2010). The PSQI is a self-reported questionnaire evaluating sleep quality during the last 1 month, consisting of 19 individual items that generate seven component scores (subjective sleep quality, sleep latency, sleep duration, habitual sleep efficiency, sleep disturbances, use of sleeping medication, and daytime dysfunction). Each sleep component had a score of 0–3 and the total score ranged from 0 to 21, corresponding to increasingly impaired sleep. Total scores ≤5, 6–10, and >10 were defined as good, general, and poor sleep, respectively, whereas each component scores 0–3 as good, general, poor, and poorer, respectively. The questionnaire also included some specific questions, such as bedtime, getup time, total time spent in bed (TIB), and nocturnal sleep duration (NSD); however, approximately one-fourth of the participants did not provide these data.

### Clinical Assessments

Detailed history taking and examination were performed for all subjects, including the evaluations of demographic and clinical characteristics (i.e., age, sex, educational level, body mass index [BMI], disease onset age, disease duration, and daily levodopa equivalent dose [LEDD]), disease severity, motor subtypes, and motor and NMSs. Trained researchers performed all examinations.

Early onset PD (EOPD) and late-onset PD (LOPD) were defined as the disease onset age before and after 50 years of age, respectively. Additionally, tremor dominant (TD) and postural instability gait disorder (PIGD) were divided based on the ratio of TD to PIGD scores: patients with ratios >1.15 were classified into TD, those with ratios <0.90 to the PIGD, and patients with ratios among 0.90–1.15 were classified as “undetermined” (Stebbins et al., 2013). LEDD was calculated according to the common conversion formulae (Tomlinson et al., 2010), and almost all patients with PD were on the medication for their condition at the time of evaluation.

Motor symptoms were measured using the Hoehn and Yahr (H&Y) stage (Goetz et al., 2004), as well as the Movement Disorders Society Unified Parkinson's Disease Rating Scale (MDS-UPDRS) part II and III (Goetz et al., 2008), whereas the quality of life was assessed using the 39-Item Parkinson's Disease Questionnaire (PDQ-39) (Jenkinson et al., 1997). The MDS-UPDRS-IV was applied to assess PD complications, and the Freezing of Gait Questionnaire (FOG-Q) was used to evaluate FOG. In addition, NMSs were evaluated using various scales (van Wamelen et al., 2021). Specifically, the MDS-UPDRS-I and Non-Motor Symptoms Scale (NMSS) were used for NMS burden; cognitive function was examined using the Mini-Mental State Examination (MMSE) and Montreal Cognitive Assessment (MoCA); Hamilton Rating Scale for Depression (HAMD) and Anxiety (HAMA) were used to evaluate mood symptoms; Scales for Outcomes in Parkinson's Disease-Autonomic (SCOPA-AUT) was used to assess autonomic dysfunction; and the Apathy Scale (AS) was used for apathy evaluation.

### Statistical Analyses

Differences in demographic characteristics between the good, general, and poor sleep groups were examined using the Mann–Whitney *U* test (continuous variables) and χ^2^ test (categorical variables). Spearman's rank correlations were used to explore the associations between sleep parameters (sleep components and times) and the clinical characteristics of PD. Furthermore, non-linear regression analyses using the quadratic model (y = αx^2^ + βx + c) were employed to investigate the non-linear associations between sleep habits (bed time, getup time, TIB, and NSD) and PD characteristics (Hayes and Preacher, 2010). When the coefficient of the quadratic term was significantly larger (U-shaped) or smaller (reverse U-shaped) than zero, significant non-linear associations were observed. Most scales did not display a normal distribution (Kolmogorov–Smirnov test, *p* < 0.05); therefore, they were log10-transformed to approximate the normal distribution. The Bonferroni method was used for multiple corrections (for seven sleep components), except where specifically noted. Next, mediation analyses were used to evaluate whether the associations between sleep and PD severity were mediated by NMSs (Baron and Kenny, 1986). In each model, sleep characteristics were included as independent variables, and PD motor symptoms and quality of life were the dependent variables. All mediation tests were performed with 10,000 bootstrap replications.

All analyses were conducted using R (version 3.6.3) and GraphPad Prism version 8.0, and the statistical significance threshold was set at two-tailed *p* < 0.05. Bonferroni corrections were applied for multiple corrections of all sleep characteristics or PD scales.

## Results

### Participant Characteristics

**Figure 1:**
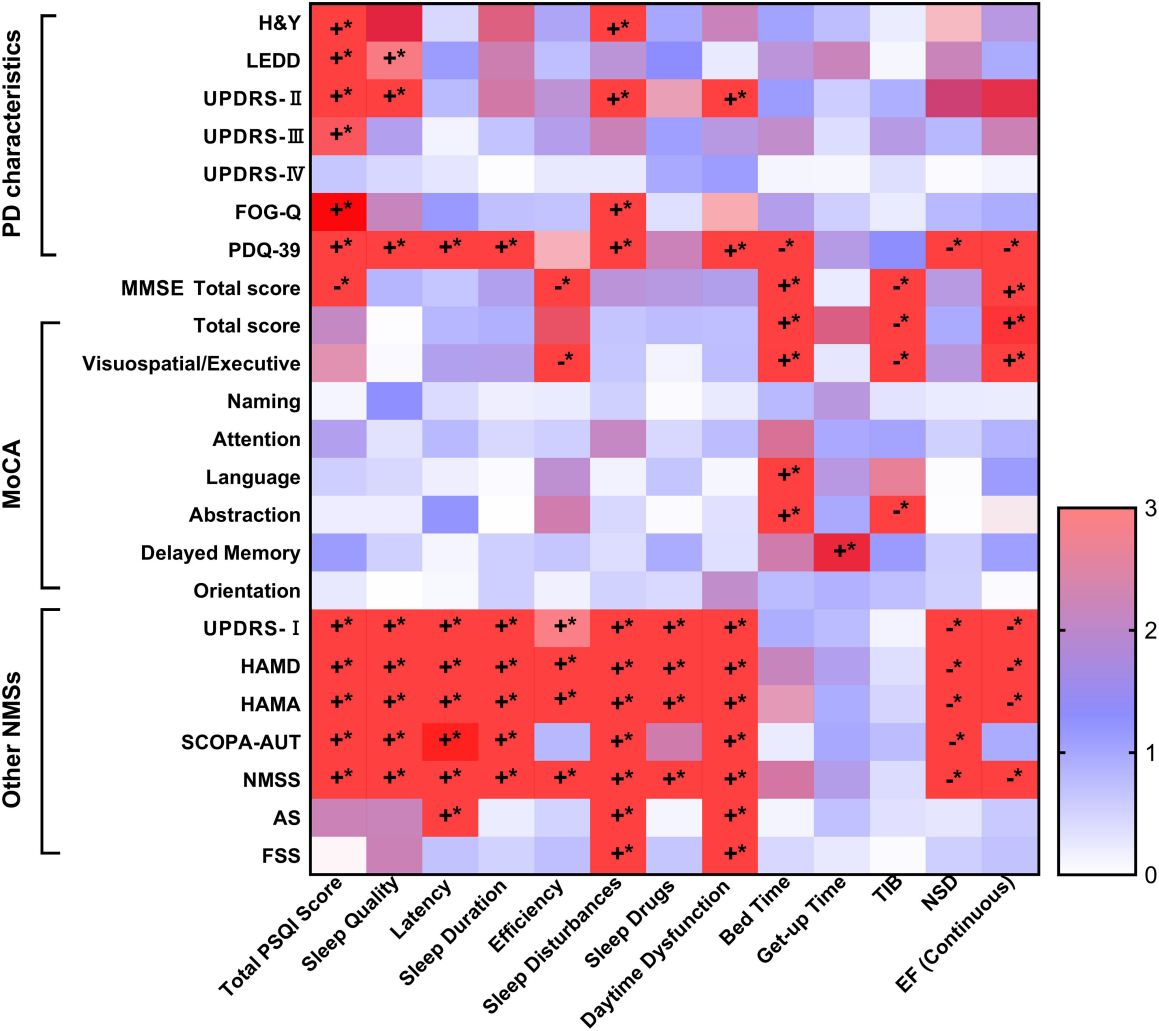
The Spearman's correlations of PSQI and PD symptoms. The sleep characteristics and habits showed significant associations with PD characteristics, as well as motor and NMSs. Specifically, the increasing total PSQI scores were related to more severe PD characteristics, including higher scores of H&Y stages, UPDRS-II and III, FOG-Q and PDQ-39, as well as higher doses of LEDD. Poorer sleep levels were also associated to worse cognitive function, though only total MMSE score, orientation, and language and praxis survived after Bonferroni correction (*p* < 0.002). Further, it was illustrated that elevated total PSQI scores correlated with more serious NMSs covered NMS burden, depression, anxiety, autonomic dysfunction, apathy, and fatigue (*p* < 0.05). *The *p*-value was survived after Bonferroni correction (*p* < 0.002). *AS*, Apathy Scale; *EF*, efficiency; *FOG-Q*, Freezing of Gait Questionnaire; *FSS*, Fatigue Severity Scale; *HAMA*, Hamilton Anxiety Rating Scale; *HAMD*, Hamilton Depression Rating Scale; *H&Y*, Hoehn-Yahr stage; *LEDD*, levodopa equivalent daily dose; *MDS-UPDRS*, Movement Disorder Society Unified Parkinson's Disease Rating Scale; *MMSE*, Mini-Mental State Examination; *MoCA*, Montreal Cognitive Assessment; *NMSS*, Non-Motor Symptoms Scales; *NSD*, nocturnal sleep duration; *PD*, Parkinson's disease; *PDQ-39*, 39-item Parkinson's Disease Questionnaire; *PSQI*, Pittsburgh Sleep Quality Index; *SCOPA-AUT*, Scale for Outcomes in PD for Autonomic Symptoms, *TIB*, total time spent in bed. The “+” was symbolized to positive correlated, while the “−” was negative correlated. The “*” was on behalf of statistically significant difference (*p* < 0.05).

The characteristics of the participants included in the present study are summarized in Table 1, whereas the detailed sleep characteristics are shown in Figure 1. A total of 328 patients with PD aged 60.5 years (*SD* = 10.1; 145 women) were enrolled. There were significant differences in disease duration among the three groups (*p* = 0.005), whereas there were no differences in age, sex, educational level, BMI, disease onset age, and subtypes (all *p* > 0.05). Additionally, poor sleep quality was associated with more severe motor and non-motor symptoms (Table 1).

**Table 1:** The characteristics of participants.

**Characteristic**	**PSQI (*n =* 328)**			***p***
	**Good**	**General**	**Poor**	
	**(*n =* 99)**	**(*n =* 147)**	**(*n =* 82)**	
**Demographic characteristics**			
Age (SD), year	59.4 (9.6)	60.9 (10.9)	61.2 (9.0)	0.222
Female%, *n* (%)	35 (35.4)	67 (45.6)	43 (52.4)	0.064
Education (*SD*), year	9.4 (4.0)	9.0 (4.7)	7.9 (4.4)	0.069
BMI (*SD*), Kg/m^2^	23.5 (3.4)	23.3 (3.2)	23.4 (3.3)	0.611
Disease onset (*SD*), year	55.4 (10.2)	55.5 (10.9)	55.6 (9.6)	0.970
Disease duration (*SD*), year	4.0 (3.8)	5.0 (4.7)	5.3 (3.9)	**0.005**
Hoehn-Yahr stage (*SD*)	1.9 (0.9)	2.2 (1.0)	2.4 (1.0)	**0.001**
LEDD (*SD*), mg	526.9 (201.8)	578.9 (265.1)	629.2 (246.4)	**0.009**
FOG-Q (*n =* 151)	4.0 (5.4)	7.0 (6.6)	9.0 (7.4)	**0.004**
**Subtypes**			
Motor (TD/PIGD/Undetermined)	41/53/5	61/79/7	26/53/3	0.554
Onset age (EOPD/LOPD/Unclear)	35/62/2	50/95/2	23/57/2	0.822
**MDS-UPDRS**				
UPDRS-I	6.0 (3.8)	9.3 (4.8)	14.2 (5.9)	**<0.001**
UPDRS-II	8.8 (6.7)	10.8 (7.8)	14.0 (8.4)	**<0.001**
UPDRS-III	30.0 (16.5)	31.8 (16.9)	35.7 (18.6)	0.091
UPDRS-IV (*n =* 293)	1.0 (2.7)	1.1 (2.6)	1.1 (2.7)	0.697
**Non-motor symptoms assessments**			
MMSE	25.6 (5.0)	25.7 (4.3)	23.8 (5.3)	**0.003**
MoCA (*n =* 300)	21.4 (6.0)	21.4 (5.7)	20.6 (5.1)	0.200
HAMD	9.2 (6.4)	12.9 (6.9)	18.0 (7.0)	**<0.001**
HAMA	7.5 (5.2)	11.3 (6.4)	17.0 (6.7)	**<0.001**
PDQ-39	31.8 (21.3)	42.7 (25.0)	53.1 (25.4)	**<0.001**
SCOPA-AUT	29.9 (5.6)	33.7 (7.4)	36.7 (7.9)	**<0.001**
NMSS	28.3 (19.1)	43.0 (29.2)	72.4 (31.4)	**<0.001**
AS (*n =* 219)	11.2 (10.5)	14.5 (11.2)	16.9 (13.9)	**0.046**
FSS (*n =* 141)	24.6 (17.7)	32.3 (18.5)	37.5 (17.0)	**0.003**

*AS, Apathy Scale; BMI, body mass index; EOPD, early-onset Parkinson's disease; FOG-Q, Freezing of Gait Questionnaire; FSS, Fatigue Severity Scale; HAMA, Hamilton Anxiety Rating Scale; HAMD, Hamilton Depression Rating Scale; LEDD, levodopa equivalent daily dose; LOPD, late-onset Parkinson's disease; UPDRS, Movement Disorder Society Unified Parkinson's Disease Rating Scale; MMSE, Mini-Mental State Examination; n, number; MoCA, Montreal Cognitive Assessment; NMSS, Non-motor symptoms Scales; PDQ-39, 39-item Parkinson's Disease Questionnaire; PIGD, postural instability gait disorder; PSQI, Pittsburgh Sleep Quality Index; SCOPA-AUT, Scale for Outcomes in PD for Autonomic Symptoms; SD, standard deviation; TD, tremor dominant. The bold values were symbolized to statistically significant difference (p < 0.05)*.

### Spearman's Correlations of PSQI Domains and PD Characteristics

Sleep characteristics and habits were significantly associated with PD characteristics as well as motor and NMSs scores (Figure 1). Specifically, the increasing total PSQI scores were related to more severe PD characteristics, including higher H&Y stage scores, UPDRS-II and III, FOG-Q, and PDQ-39, as well as higher doses of LEDD (all Spearman's *p* < 0.01). Poorer sleep levels were also associated with worse cognitive function, although only the total MMSE score, total MoCA score, and executive function survived after Bonferroni correction (*p* < 0.002). Furthermore, elevated total PSQI scores correlated with more serious NMS burden, depression, anxiety, autonomic dysfunction, apathy, and fatigue (*p* < 0.05). Additionally, different sleep components were associated with PD characteristics, as shown in Figure 1 and Supplementary Tables 1, 2.

### Non-Linear Associations of Sleep Habits and PD Characteristics

**Figure 2:**
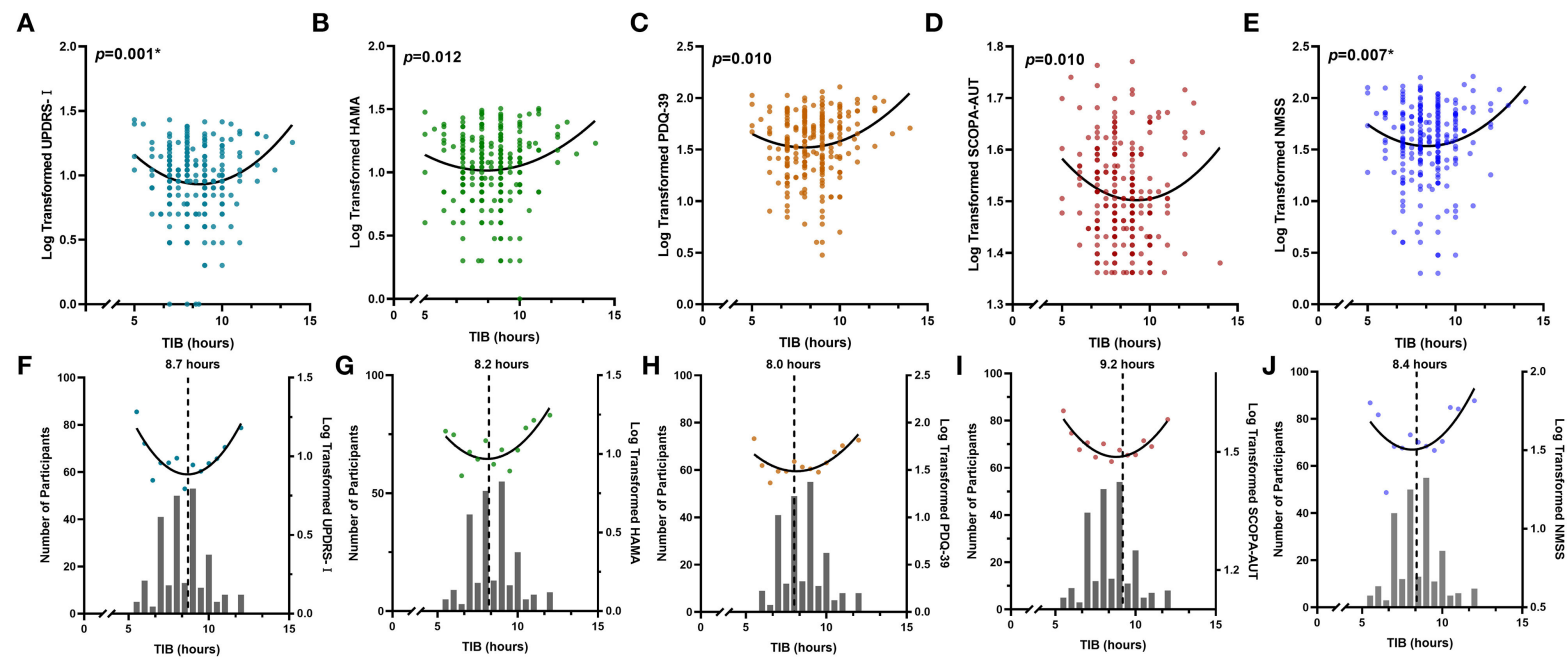
Non-linear associations between sleep habits and PD symptoms. Total time spent in bed showed reverse U-shaped associations with different NMSs, including UPDRS-I **(A,F)**, HAMA **(B,G)**, PDQ-39 **(C,H)**, SCOPA-AUT **(D,I)**, and NMSS **(E,J)**. Either insufficient or excessive TIB is associated with worse performances of NMSs. The numbers of individuals with each sleep hour are shown in the bar graph. *The *p*-value was survived after Bonferroni correction (*p* < 0.008). *HAMA*, Hamilton Anxiety Rating Scale; *MDS-UPDRS*, Movement Disorder Society Unified Parkinson's Disease Rating Scale; *NMSS*, Non-Motor Symptoms Scales; *PDQ-39*, 39-item Parkinson's Disease Questionnaire; *SCOPA-AUT*, Scale for Outcomes in PD for Autonomic Symptoms; *TIB*, total time spent in bed.

There were significant non-linear relationships between sleep habits and motor and non-motor symptoms in individuals with PD (Table 2). The reflection point for bedtime was ~21:52, associated with UPDRS-II, whereas no significant non-linear relationships were found for the getup time. The recommended TIB was ~8–9.2 h, indicating that the fitted curve reached its peak (Figure 2). Both insufficient and excessive TIB are associated with a higher NMS burden. Conversely, the reflection points were suited 6.2–6.9 h, and either fewer and more were disadvantageous for PD characteristics, suggesting that the proper sleep duration was ~6–7 h per day.

**Table 2:** The associations of PSQI parameters with PD characteristics using nonlinear regression models.

**Characteristic**	**PSQI**											
	**Bed time**			**Get-up time**			**TIB**			**NSD**		
	**β**	**α**	**Extreme point**	**β**	**α**	**Extreme point**	**β**	**α**	**Extreme point**	**β**	**α**	**Extreme point**
**Motor symptoms**
UPDRS-II	**−0.543**	**0.028**	**21:52^b^**	ns	ns	ns	−0.193	0.012	7.9h^a^	ns	ns	ns
UPDRS-III	−0.362	0.017	22:24^a^	ns	ns	ns	ns	ns	ns	ns	ns	ns
UPDRS-IV	ns	ns	ns	ns	ns	ns	ns	ns	ns	ns	ns	ns
FOG-Q	ns	ns	ns	ns	ns	ns	ns	ns	ns	**−2.287**	**0.186**	**6.2h^b^**
**Non-Motor Symptoms**
UPDRS-I	ns	ns	ns	ns	ns	ns	**−0.289**	**0.017**	8.7h^c^^*^	**−0.932**	**0.068**	**6.9h^b^**
MMSE	ns	ns	ns	ns	ns	ns	ns	ns	ns	ns	ns	ns
MOCA	ns	ns	ns	ns	ns	ns	ns	ns	ns	ns	ns	ns
HAMD	ns	ns	ns	ns	ns	ns	ns	ns	ns	ns	ns	ns
HAMA	ns	ns	ns	ns	ns	ns	**−0.196**	**0.012**	**8.2h^b^**	ns	ns	ns
PDQ-39	−0.398	0.019	10:38^a^	ns	ns	ns	**−0.235**	**0.015**	**8.0h^b^**	−0.903	0.068	6.6h^a^
SCOPA-AUT	ns	ns	ns	ns	ns	ns	**−0.083**	**0.004**	**9.2h^b^**	ns	ns	ns
NMSS	ns	ns	ns	ns	ns	ns	**−0.309**	**0.018**	8.4h^c^^*^	**−1.741**	**0.133**	6.6h^c^^*^
AS	ns	ns	ns	ns	ns	ns	ns	ns	ns	ns	ns	ns
FSS	ns	ns	ns	ns	ns	ns	−0.204	0.012	8.7h^a^	ns	ns	ns

a *p <0.10 (p for trend)*.

b *p <0.05*.

c *p <0.01*.

* *The p-value was survived after Bonferroni correction (p < 0.008)*.

*AS, Apathy Scale; FOG-Q, Freezing of Gait Questionnaire; FSS, Fatigue Severity Scale; HAMA, Hamilton Anxiety Rating Scale; HAMD, Hamilton Depression Rating Scale; UPDRS, Movement Disorder Society Unified Parkinson's Disease Rating Scale; MMSE, Mini-Mental State Examination; MoCA, Montreal Cognitive Assessment; NMSS, Non-motor symptoms Scales; NSD, nocturnal sleep duration; PDQ-39, 39-item Parkinson's Disease Questionnaire; PSQI, Pittsburgh Sleep Quality Index; SCOPA-AUT, Scale for Outcomes in PD for Autonomic Symptoms; TIB, total time spent in bed. The bold values were symbolized to statistically significant difference (p < 0.05)*.

### Mediation Analyses

**Figure 3:**
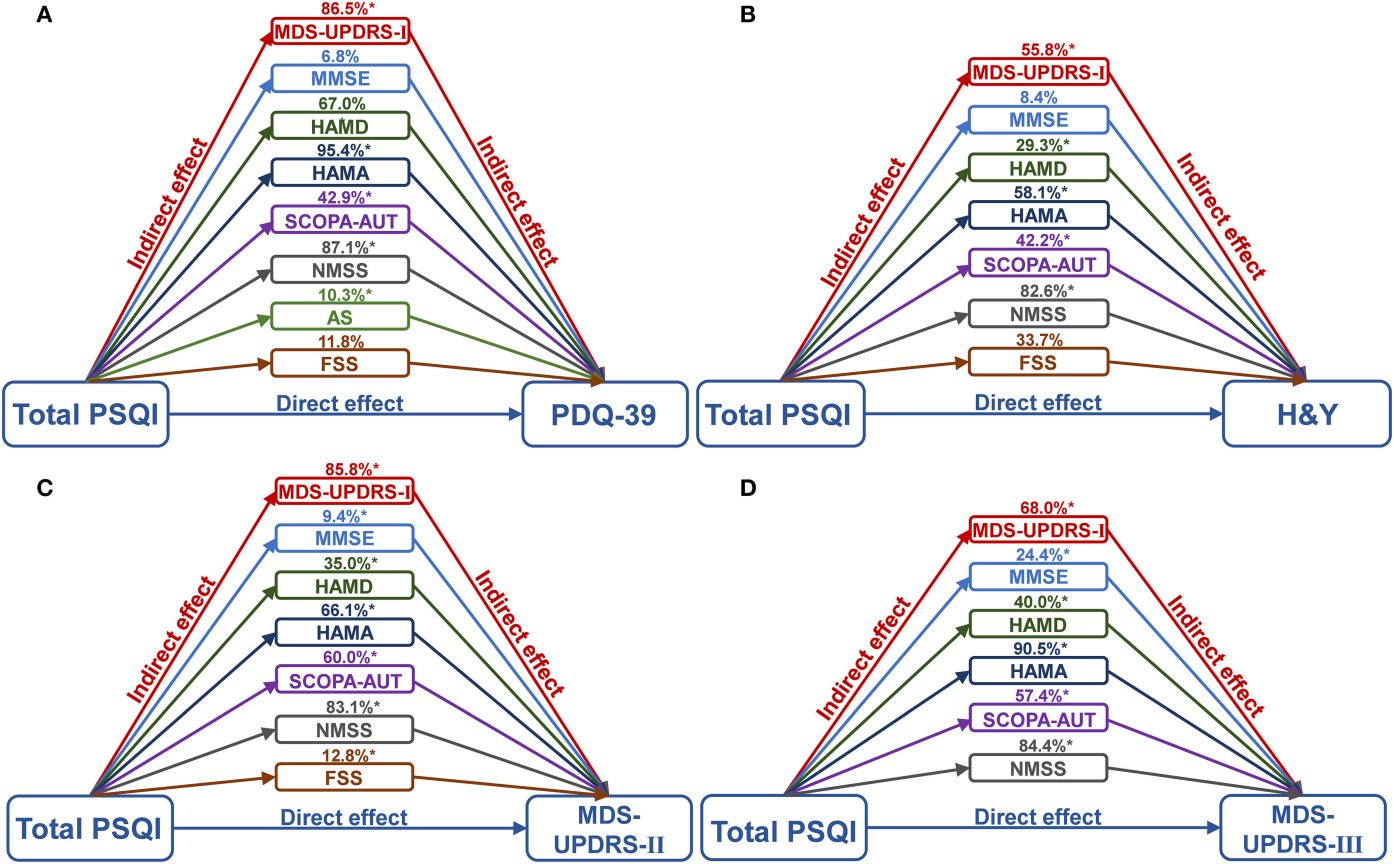
Mediation analyses of NMSs with global sleep quality and PD. It was found that various scales of NMSs, consisting of MDS-UPDRS-I, MMSE, HAMD, HAMA, SCOPA-AUT, NMSS, AS, and FSS, could mediate the effect of quality of life **(A)**, and the severity of PD, including H&Y stages **(B)**, MDS-UPDRS II **(C)** and III **(D)** with a range of 6.8–95.4%. *The *p*-value was survived after Bonferroni correction (*p* < 0.001). *AS*, Apathy Scale; *FSS*, Fatigue Severity Scale; *HAMA*, Hamilton Anxiety Rating Scale; *HAMD*, Hamilton Depression Rating Scale; *MDS-UPDRS*, Movement Disorder Society Unified Parkinson's Disease Rating Scale; *MMSE*, Mini-Mental State Examination; n, number; *MoCA*, Montreal Cognitive Assessment; *NMSS*, Non-Motor Symptoms Scales; *PD*, Parkinson's disease; *PDQ-39*, 39-item Parkinson's Disease Questionnaire; *PSQI*, Pittsburgh Sleep Quality Index; *SCOPA-AUT*, Scale for Outcomes in PD for Autonomic Symptoms.

Mediation analyses were performed to investigate whether NMSs contributed to the quality of life and severity of PD as assessed by sleep levels (Figure 3 and Supplementary Tables 3–6). We found that various scales of NMSs, consisting of the MDS-UPDRS-I, MMSE, HAMD, HAMA, SCOPA-AUT, NMSS, AS, and FSS, could mediate the effect of quality of life (PDQ-39) and the severity of PD (H&Y stages, MDS-UPDRS II and III) with a range of 6.8–95.4%. Anxiety, depression, and NMS burden showed leading mediated effects, whereas cognition and fatigue did not survive after the Bonferroni correction (*p* < 0.001).

## Discussion

This study comprehensively evaluated the association of sleep characteristics and habits with motor symptoms and NMSs in patients with PD using a Chinese cohort. The primary results showed that poorer sleep levels were linked to more severe motor and non-motor symptoms in individuals with PD. Notably, we found several reflection points that showed the best bedtime, as well as the appropriate TIB and NSD, which were most beneficial to PD symptoms. Equally, we discovered that the associations between sleep quality and PD severity, quality of life, and motor symptoms were mediated by a number of NMSs to varying degrees, which provides new insights for clinical practice to intervene in PD deterioration with sleep disorders. Our findings also highlight the importance of increasing the awareness of sleep problems in patients with PD.

The PSQI was chosen to evaluate global sleep quality because it has been generally used for the occurrence of sleep disturbances in PD populations with good sensitivity and specificity (Pal et al., 2004). The PSQI has been reported to be sensitive to subjective sleep and therefore can be used to screen for the presence of sleep alteration and to rate the severity ulteriorly. Consistent with our results, previous studies have shown that poor sleep quality and efficiency are the promising markers of prodromal PD, and that an increased number of sleep disturbances and poor nighttime sleep are positively correlated with PD-related disability (Mao et al., 2018; Lysen et al., 2019). It is suggested that lesions of the lower brainstem expand and sleep disorders become more pronounced with PD deterioration (Braak et al., 2004). Motor symptoms aggravate the condition of patients, and they often combine with advanced multi-autonomic dysfunctions such as nocturia, nightmares, and restless leg syndrome, which further affect sleep. Since the maintenance of the sleep-wake cycle is closely related to dopamine (Lima, 2013), patients with PD with advanced motor symptoms tend to experience aggravation of sleep disturbances, which may be related to the degeneration of the dopaminergic system in the nigrostriatal pathway. Sleep disturbances may also contribute to the progression of PD symptoms (Lauretti et al., 2017). These nocturnal sleep problems can be aggravated by discomfort resulting from nighttime hypokinesia, particularly impaired bed turning; meanwhile, pain and other sensations caused by PD may also negatively influence sleep.

One novelty of this study is that we observed a U-shaped relationship between sleep habits and symptoms of PD. Bed time has shown positive non-linear associations with motor symptoms in patients with PD, whereas either excessive or insufficient TIB and NSD are unprofitable for the quality of life and NMSs. Several studies have reported that excessive sleep duration increases PD risk (Zhang et al., 2021), and others have found that too little nocturnal sleep duration increases the risk of motor symptoms (Lysen et al., 2019). Therefore, inadequate TIB and NSD could possibly lead to worse motor and NMS burden and even the deterioration of PD, which is consistent with our results. Likewise, disruptions to the sleep–wake cycle have been associated with inefficient metabolic clearance and increased oxidative stress in the central nervous system, which leads to excessive accumulation of alpha-synuclein and induction of neuronal loss, thus accelerating the pathogenesis and progression of PD. A cross-sectional study has proven that poor sleep, such as insufficient or excessive sleep duration and unsuitable sleep time, is associated with lower levels of cerebrospinal fluid alpha-synuclein in non-PD individuals (Wang et al., 2020). These U-shaped associations between sleep duration and PD can be explained from the perspectives of sleep quality and efficiency. As recommended, we claim that the optimum bed time, TIB, and NSD are approximately 22 o'clock, 8–9, and 6–7 h, respectively. Conversely, the overnight sleep for healthy age is often fragmented and lasts for <6.0–7.5 h due to the changes in sleep and circadian rhythms with aging (Gulia and Kumar, 2018). Compared with healthy elders, patients with PD have more serious sleep problems as a disorder of sleep maintenance, but also as a disorder of sleep onset or early morning awakening due to motor and non-motor symptoms. Therefore, it is more difficult, but important, for them to obtain sufficient sleep.

The impairment of sleep rhythm significantly impacts the quality of life in PD, including autonomic, cognitive, and psychiatric functions in patients with PD, in turn worsening motor manifestations and affective symptoms (Videnovic et al., 2014a). Additionally, non-motor problems, such as nocturia, related pain, and neuropsychiatric symptoms, often give rise to sleep fragmentation. The generation and maintenance of the sleep–wake cycle depend on the central neurotransmitter systems that mainly involve 5-hydroxytryptamine, norepinephrine, and acetylcholine, and studies have indicated that these monoaminergic neurotransmitters play the important roles in regulating circuits related to emotion, psychology, stress, and cognitive performance (Thobois et al., 2017). The dynamic equilibrium and interaction of neurotransmitters determine normal functions of the circadian rhythm, and the neurons undergo degeneration and death to varying degrees in patients with PD, which can explain the significant correlation between NMSs and sleep disturbances. Another novel finding of this study is that non-motor complications, such as cognitive decline, depression, anxiety, autonomic dysfunction, apathy, and fatigue, partly mediate the impact of global sleep quality on PD severity, quality of life, and motor symptoms. These mediating effects varied from 10 to 90%, suggesting that NMSs and sleep disturbances are not independently linked to PD severity. Therefore, psychiatric symptoms contributed the most. These may be attributed to multi-system impairments, including the nigrostriatal dopaminergic system (Pfeiffer, 2016), and lead to the assumption that multiple coexisting conditions, such as motor and NMSs, contribute to sleep disorders (Kurtis et al., 2013). However, cognition and fatigue have less mediating effects, which suggests the independent roles in sleep.

Cognitive impairment is one of the most epidemic NMSs of PD, with a prevalence of 30–40%, and worse sleep efficiencies in PD subjects tend to be significantly associated with working memory and verbal memory consolidation (Emre, 2003). Our results also correlated with orientation and executive function. Anxiety and depression are the important factors that affect sleep status in patients with PD, and emotion generation and regulation are influenced by alterations in the frontal limbic systems (Palmer and Alfano, 2017). The relationships between sleep disturbances and emotional symptoms are bidirectional and can give rise to vicious circles for PD individuals, in which both reinforce each other (Rutten et al., 2017). A study reported that reduced melatonin secretion results in a 4-fold decrease in circulating melatonin levels in patients with PD (Videnovic et al., 2014b), often associated with cognitive decline and psychiatric disorders. Additionally, premature occurrence of apathy symptoms may forebode the existence of sleep disorders with procession NMSs, and clinical diagnosis and treatment should pay more attention to apathy. Autonomic failure and fatigue are the pervasive problems associated with PD (Suzuki et al., 2014; Siciliano et al., 2018). Gastrointestinal distress, such as constipation and defecatory dysfunction, causes discomfort and an unpredictable need for toilets at night. Urinary symptoms such as nocturia are common among patients with PD and may also contribute to disrupted sleep as well (Bjørnarå et al., 2014). Sleep disturbances thus seem complex and likely multifactorial, and comorbidities probably play roles. However, sleep disorders in PD have not received sufficient attention and are consequently under-diagnosed and untreated. The treatment of sleep problems will likely improve NMSs but also postpone PD progression. It can potentially reduce the overall disability and thereby improve the lives of patients and their caregivers (Leroi et al., 2010).

Furthermore, sleep disorders were more likely to be positively correlated with higher LEDD in our study and previous studies (Verbaan et al., 2008; Antczak et al., 2013). Levodopa treatment is believed to improve sleep efficiency with reduced sleep latency by improving motor scores (Ferreira et al., 2014) and is also able to improve sleep quality partly by reducing night-time dyskinesia or tremor which severely interferes with normal sleep (Poewe et al., 2007). However, these drugs, particularly at higher doses, may cause insomnia and excessive daytime sleepiness, with a sudden onset of sleep attacks. One hypothesis is that poor sleep complainants receive higher doses of levodopa than good sleepers, rather than higher doses of levodopa, leading to poor sleep (Mao et al., 2017). Additionally, antiparkinsonian drugs do not work at midnight due to the levodopa doses in the blood, leading to various PD symptoms, and can cause vivid dreams, hallucinations, and paranoia, particularly at night. Our results showing that the associations between sleep and LEDD do not exist after Bonferroni correction have verified the complexities of levodopa treatment on sleep, which needs further exploration.

This study has several strengths and limitations. Our analyses systematically evaluated the sleep disorders and global PD characteristics and found worthy results such as optimal sleep habits for patients with PD. Meanwhile, the current findings stress the mediating effect of NMSs between sleep disorders and PD symptoms and quality of life, enabling the development of comprehensive therapeutic approaches. Conversely, the PSQI is a self-reported scale that does not reflect objective sleep quality, and there are different sleep disorders in PD beyond insomnia, such as apnea, rapid eye movement sleep behavior disorder, and excessive daytime sleepiness which could not be disentangled using the PSQI or any single scale that is needed to select objective measures such as polysomnography for sleep monitoring. In addition, this study only sought to propose strong hypotheses about the potential roles of sleep disorders in PD and will be examined in future longitudinal studies.

## Conclusions

In summary, our cross-sectional study indicated a close relationship between sleep characteristics and the burden of PD symptoms in patients with PD. Poor sleep quality and efficiency as well as insufficient or excessive sleep duration are associated with more severe PD symptoms, including PD stages, motor symptoms, NMSs, and quality of life. Therefore, our findings provide new insights into the monitoring and management of sleep and PD, which need to be further explored in the future studies.

## Data Availability Statement

The raw data supporting the conclusions of this article will be made available by the authors, without undue reservation.

## Ethics Statement

The studies involving human participants were reviewed and approved by Tongji Hospital, Tongji College of Medicine, Huazhong University of Science and Technology, Wuhan, China. The patients/participants provided their written informed consent to participate in this study.

## Author Contributions

XZ and QY designed and conceptualized study. QY, CY-P, LJ-T, LJ-Y, QQ-X, WD-L, and ZJ-W conducted the study. QY, MZ-J, XY-J, MZ, and XZ analyzed and extracted the data. QY, CY-P, and XZ wrote the first draft of the manuscript. All authors reviewed the manuscript.

## Funding

This work was funded by the National Natural Science Foundation of China (Grant Nos. 81771376 and 91849121).

## Conflict of Interest

The authors declare that the research was conducted in the absence of any commercial or financial relationships that could be construed as a potential conflict of interest.

## Publisher's Note

All claims expressed in this article are solely those of the authors and do not necessarily represent those of their affiliated organizations, or those of the publisher, the editors and the reviewers. Any product that may be evaluated in this article, or claim that may be made by its manufacturer, is not guaranteed or endorsed by the publisher.
