# Supplementary material for: Worse Sleep Quality Aggravates the Motor and Non-Motor Symptoms in Parkinson's Disease
Source: Front Aging Neurosci. 2022 Jun 10;14:887094. doi: 10.3389/fnagi.2022.887094 (PMC9226540; doi:10.3389/fnagi.2022.887094)
Supplement: Supplementary file 1 [file Data_Sheet_1.docx]

Supplementary Material

# Supplementary Figures and Tables

## Supplementary Figures


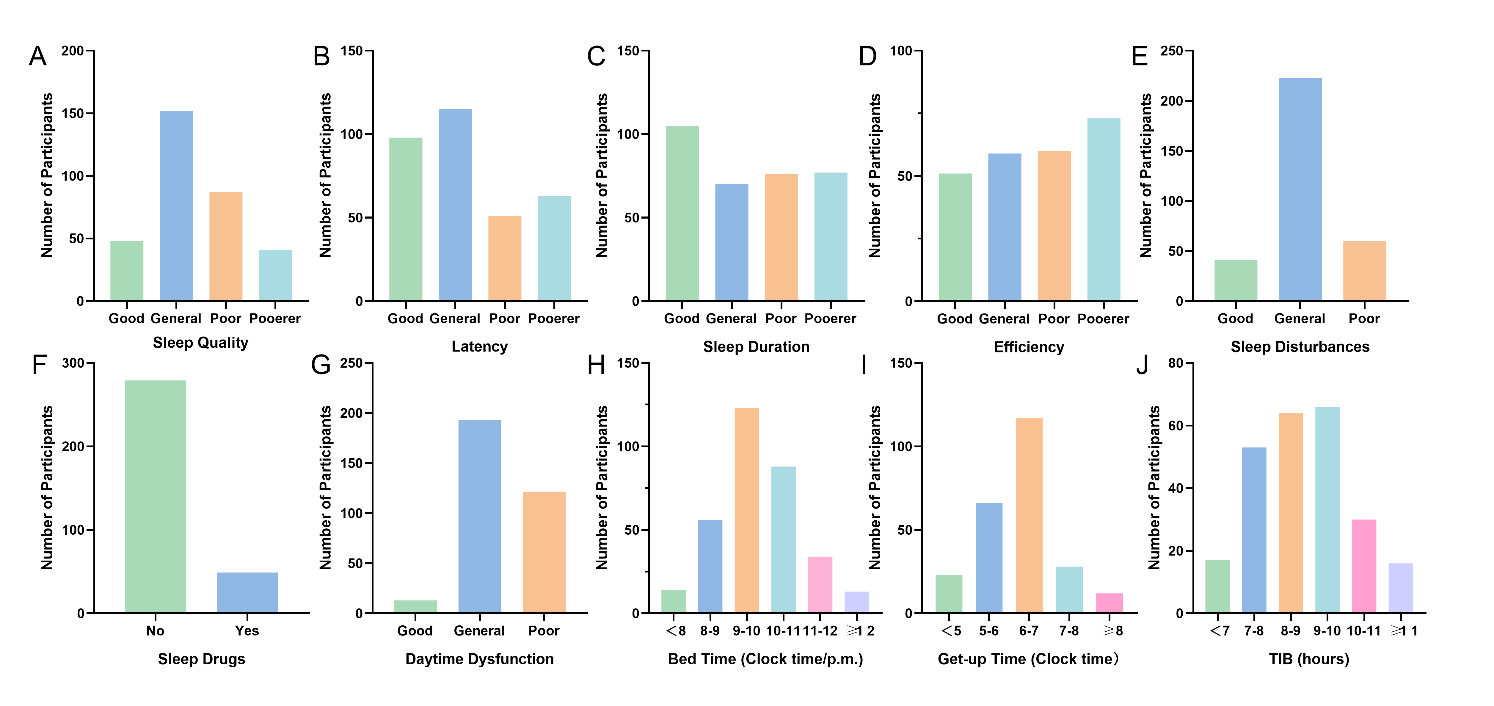


**Supplementary Figure 1.** **The sleep characteristics information of PSQI in PD participants.**

The column charts of sleep characteristics summarized number of participants from included individuals. The groups of sleep characteristics are arranged left-right hierarchically on the sleep quality from good to poorer, as well as the sleep habits were demonstrated according to various time quantum. The sleep characteristics included sleep quality (0, 1, 2, 3 points) (A), sleep latency (≤15 minutes, 16-30 minutes, 31-60 minutes, >60 minutes) (B), sleep duration (>7 hours, 6-7 hours, 5-6 hours, ≤5 hours) (C), sleep efficiency (>85%, 75-84%, 65-74%, ≤65%) (D), sleep disturbances (0, 1, 2-3 points) (E), use of sleeping medication (use, not use) (F), and daytime dysfunction (0, 1, 2 points) (G), as well as sleep habits included bed time (H), get-up time (I) and TIB (J). Abbreviations: PD, Parkinson’s disease; PSQI, Pittsburgh Sleep Quality Index; TIB, Total Time Spent in Bed.

## Supplementary Tables

**Supplementary Table 1. The Spearman correlations between PSQI and PD.**

| Characteristic | Total Score | | Factor 1 | | Factor 2 | | Factor 3 | | Factor 4 | | Factor 5 | | Factor 6 | | Factor 7 | |
| --- | --- | --- | --- | --- | --- | --- | --- | --- | --- | --- | --- | --- | --- | --- | --- | --- |
|  | *ρ* | *p* | *ρ* | *p* | *ρ* | *p* | *ρ* | *p* | *ρ* | *p* | *ρ* | *p* | *ρ* | *p* | *ρ* | *p* |
| PD Characteristics |  |  |  |  |  |  |  |  |  |  |  |  |  |  |  |  |
| H&Y | **0.191** | **0.000^*^** | **0.174** | **0.003** | 0.098 | 0.366 | **0.142** | **0.004** | **0.084** | **0.041** | **0.127** | **0.000^*^** | 0.108 | 0.102 | **0.031** | **0.012** |
| LEDD | **0.240** | **0.001^*^** | **0.165** | **0.002^*^** | 0.050 | 0.075 | **0.157** | **0.010** | 0.131 | 0.190 | **0.214** | **0.023** | 0.091 | 0.051 | 0.139 | 0.576 |
| UPDRS-Ⅱ | **0.296** | **0.000****^*^** | **0.243** | **0.000****^*^** | 0.077 | 0.164 | **0.146** | **0.008** | **0.148** | **0.021** | **0.274** | **0.000****^*^** | **0.155** | **0.005** | **0.255** | **0.000^*^** |
| UPDRS-Ⅲ | **0.179** | **0.001^*^** | **0.120** | **0.034** | -0.020 | 0.730 | 0.071 | 0.208 | **0.140** | **0.033** | **0.143** | **0.011** | 0.099 | 0.081 | **0.125** | **0.028** |
| UPDRS-Ⅳ | 0.070 | 0.233 | 0.055 | 0.352 | 0.039 | 0.505 | 0.004 | 0.950 | -0.04 | 0.553 | 0.035 | 0.556 | 0.095 | 0.105 | 0.104 | 0.077 |
| FOG-Q | **0.262** | **0.001^*^** | **0.201** | **0.013** | 0.148 | 0.070 | 0.106 | 0.194 | 0.113 | 0.212 | **0.331** | **0.000^*^** | 0.065 | 0.431 | **0.251** | **0.002^*^** |
| PDQ-39 | **0.366** | **0.000^*^** | **0.307** | **0.000^*^** | **0.222** | **0.000^*^** | **0.197** | **0.000****^*^** | **0.195** | **0.002** | **0.289** | **0.000^*^** | **0.139** | **0.012** | **0.240** | **0.000^*^** |
| MMSE |  |  |  |  |  |  |  |  |  |  |  |  |  |  |  |  |
| Total score | **-0.213** | **0.000^*^** | -0.081 | 0.147 | -0.069 | 0.218 | **-0.116** | **0.036** | **-0.241** | **0.000^*^** | **-0.128** | **0.022** | **-0.123** | **0.027** | **-0.118** | **0.034** |
| Orientation | **-0.197** | **0.000^*^** | -0.107 | 0.054 | -0.093 | 0.093 | -0.068 | 0.224 | **-0.187** | **0.004** | **-0.110** | **0.048** | **-0.119** | **0.032** | **-0.150** | **0.007** |
| Registration | 0.051 | 0.362 | 0.018 | 0.744 | 0.041 | 0.459 | 0.062 | 0.264 | -0.061 | 0.344 | 0.021 | 0.710 | **0.117** | **0.035** | 0.001 | 0.992 |
| Attention and Calculation | **-0.145** | **0.009** | -0.054 | 0.333 | -0.054 | 0.336 | **-0.094** | **0.091** | **-0.145** | **0.024** | -0.087 | 0.119 | -0.095 | 0.088 | -0.106 | 0.056 |
| Recall | **-0.138** | **0.013** | -0.069 | 0.217 | -0.032 | 0.566 | -0.081 | 0.143 | **-0.167** | **0.009** | -0.065 | 0.246 | -0.09 | 0.106 | -0.028 | 0.619 |
| Language and Praxis | **-0.189** | **0.001^*^** | -0.033 | 0.559 | -0.078 | 0.163 | **-0.135** | **0.015** | **-0.233** | **0.000^*^** | -0.099 | 0.075 | -0.04 | 0.469 | -0.101 | 0.068 |
| MoCA |  |  |  |  |  |  |  |  |  |  |  |  |  |  |  |  |
| Total score | **-0.141** | **0.015** | -0.002 | 0.966 | -0.083 | 0.152 | -0.088 | 0.127 | **-0.201** | **0.003** | -0.072 | 0.217 | -0.078 | 0.176 | -0.077 | 0.186 |
| Visuospatial/Executive | **-0.158** | **0.006** | 0.010 | 0.861 | **-0.121** | **0.036** | **-0.123** | **0.033** | **-0.246** | **0.000****^*^** | -0.070 | 0.231 | -0.019 | 0.747 | -0.078 | 0.179 |
| Naming | 0.016 | 0.785 | 0.111 | 0.054 | 0.049 | 0.398 | -0.027 | 0.647 | -0.036 | 0.598 | -0.061 | 0.293 | -0.008 | 0.886 | -0.031 | 0.589 |
| Attention | **-0.122** | **0.035** | -0.041 | 0.475 | -0.081 | 0.160 | -0.053 | 0.359 | -0.073 | 0.277 | **-0.142** | **0.014** | -0.052 | 0.366 | -0.079 | 0.176 |
| Language | -0.063 | 0.280 | 0.052 | 0.367 | -0.026 | 0.655 | -0.007 | 0.899 | **-0.156** | **0.020** | -0.02 | 0.726 | -0.072 | 0.214 | -0.015 | 0.792 |
| Abstraction | -0.028 | 0.629 | 0.028 | 0.631 | -0.109 | 0.061 | 0.000 | 0.998 | **-0.173** | **0.010** | 0.053 | 0.359 | -0.006 | 0.918 | 0.043 | 0.459 |
| Delayed Memory | -0.103 | 0.076 | -0.061 | 0.295 | -0.017 | 0.773 | -0.063 | 0.277 | -0.081 | 0.227 | -0.048 | 0.413 | -0.092 | 0.111 | -0.045 | 0.436 |
| Orientation | -0.034 | 0.557 | 0.001 | 0.988 | 0.012 | 0.840 | 0.061 | 0.291 | -0.027 | 0.688 | -0.059 | 0.313 | -0.052 | 0.374 | **-0.138** | **0.017** |
| Other Non-Motor Symptoms |  |  |  |  |  |  |  |  |  |  |  |  |  |  |  |  |
| UPDRS-Ⅰ | **0.542** | **0.000^*^** | **0.488** | **0.000^*^** | **0.325** | **0.000^*^** | **0.337** | **0.000^*^** | **0.204** | **0.001^*^** | **0.430** | **0.000****^*^** | **0.256** | **0.000^*^** | **0.404** | **0.000^*^** |
| HAMD | **0.459** | **0.000^*^** | **0.469** | **0.000^*^** | **0.375** | **0.000^*^** | **0.268** | **0.000^*^** | **0.226** | **0.000****^*^** | **0.229** | **0.000^*^** | **0.240** | **0.000^*^** | **0.233** | **0.000^*^** |
| HAMA | **0.553** | **0.000^*^** | **0.514** | **0.000^*^** | **0.368** | **0.000^*^** | **0.307** | **0.000^*^** | **0.252** | **0.000^*^** | **0.340** | **0.000^*^** | **0.288** | **0.000^*^** | **0.358** | **0.000^*^** |
| SCOPA-AUT | **0.367** | **0.000^*^** | **0.319** | **0.000^*^** | **0.182** | **0.001^*^** | **0.225** | **0.000^*^** | 0.092 | 0.156 | **0.332** | **0.000^*^** | **0.145** | **0.009** | **0.363** | **0.000^*^** |
| NMSS | **0.529** | **0.000^*^** | **0.446** | **0.000^*^** | **0.258** | **0.000^*^** | **0.344** | **0.000^*^** | **0.264** | **0.000^*^** | **0.355** | **0.000^*^** | **0.252** | **0.000^*^** | **0.351** | **0.000^*^** |
| AS | **0.170** | **0.011** | **0.169** | **0.012** | **0.271** | **0.000^*^** | 0.034 | 0.622 | 0.078 | 0.316 | **0.249** | **0.000^*^** | 0.018 | 0.789 | **0.274** | **0.000^*^** |
| FSS | **0.245** | **0.003** | **0.213** | **0.011** | 0.107 | 0.207 | 0.086 | 0.313 | 0.115 | 0.190 | **0.304** | **0.000^*^** | 0.102 | 0.227 | **0.430** | **0.000^*^** |

**^*^** The *p*-value was survived after Bonferroni correction (*p*<0.002).

Factor 1, sleep quality; Factor 2, sleep latency; Factor 3, sleep duration; Factor 4, sleep efficiency; Factor 5, sleep disturbances; Factor 6, use of sleeping medication; Factor 7, daytime dysfunction.

*Abbreviations*: *AS*, Apathy Scale; *EF*, Effienciey; *FOG-Q*, Freezing of Gait Questionnaire; *FSS*, Fatigue Severity Scale; *HAMA*, Hamilton Anxiety Rating Scale; *HAMD*, Hamilton Depression Rating Scale; *H&Y,* Hoehn-Yahr stage; *LEDD*, levodopa equivalent daily dose; *UPDRS*, Movement Disorder Society Unified Parkinson’s Disease Rating Scale; *MMSE*, Mini-Mental State Examination; *MoCA*, Montreal Cognitive Assessment; *NMSS*, Non-motor symptoms Scales; *PDQ-39*, 39-item Parkinson’s Disease Questionnaire; *PSQI*, Pittsburgh Sleep Quality Index; *SCOPA-AUT*, Scale for Outcomes in PD for Autonomic Symptoms.

**Supplementary Table 2. The Spearman correlations between sleep habits and PD.**

| Characteristic | Bed time | | Get-up time | | TIB | | NSD | | EF (Continuous) | |
| --- | --- | --- | --- | --- | --- | --- | --- | --- | --- | --- |
|  | *ρ* | *p* | *ρ* | *p* | *ρ* | *p* | *ρ* | *p* | *ρ* | *p* |
| PD Characteristics |  |  |  |  |  |  |  |  |  |  |
| H&Y | -0.126 | 0.087 | -0.161 | 0.184 | -0.016 | 0.610 | **-0.139** | **0.003** | **-0.101** | **0.027** |
| LEDD | **-0.095** | **0.023** | **-0.086** | **0.012** | 0.033 | 0.808 | **-0.165** | **0.012** | -0.142 | 0.115 |
| UPDRS-Ⅱ | -0.099 | 0.076 | -0.072 | 0.271 | 0.099 | 0.124 | **-0.153** | **0.006** | **-0.192** | **0.003** |
| UPDRS-Ⅲ | **-0.136** | **0.017** | -0.054 | 0.418 | 0.143 | 0.028 | -0.081 | 0.156 | **-0.167** | **0.011** |
| UPDRS-Ⅳ | -0.099 | 0.781 | -0.072 | 0.791 | 0.099 | 0.431 | -0.153 | 0.905 | -0.192 | 0.738 |
| FOG-Q | **-0.177** | **0.032** | -0.100 | 0.277 | 0.048 | 0.592 | -0.115 | 0.161 | -0.141 | 0.116 |
| PDQ-39 | **-0.196** | **0.000****^*^** | **-0.142** | **0.029** | 0.124 | 0.052 | **-0.204** | **0.000****^*^** | **-0.238** | **0.000^*^** |
| MMSE |  |  |  |  |  |  |  |  |  |  |
| Total score | **0.209** | **0.000****^*^** | 0.034 | 0.605 | **-0.211** | **0.001^*^** | **0.122** | **0.028** | **0.257** | **0.000^*^** |
| Orientation | **0.144** | **0.010** | -0.007 | 0.918 | **-0.164** | **0.010** | 0.071 | 0.205 | **0.201** | **0.002^*^** |
| Registration | **0.160** | **0.004** | 0.026 | 0.693 | -0.108 | 0.091 | -0.066 | 0.235 | 0.082 | 0.205 |
| Attention and Calculation | **0.141** | **0.012** | 0.087 | 0.181 | **-0.134** | **0.036** | 0.102 | 0.067 | **0.162** | **0.012** |
| Recall | **0.123** | **0.028** | -0.057 | 0.381 | **-0.127** | **0.048** | 0.080 | 0.150 | **0.160** | **0.013** |
| Language and Praxis | **0.205** | **0.000^*^** | 0.032 | 0.622 | **-0.182** | **0.004** | **0.137** | **0.014** | **0.257** | **0.000^*^** |
| MoCA |  |  |  |  |  |  |  |  |  |  |
| Total score | **0.275** | **0.000^*^** | **0.189** | **0.005** | **-0.235** | **0.000^*^** | 0.093 | 0.110 | **0.217** | **0.001^*^** |
| Visuospatial/Executive | **0.222** | **0.000^*^** | 0.044 | 0.519 | **-0.223** | **0.001^*^** | **0.129** | **0.026** | **0.260** | **0.000^*^** |
| Naming | 0.082 | 0.161 | **0.149** | **0.026** | -0.046 | 0.488 | 0.030 | 0.602 | 0.034 | 0.615 |
| Attention | **0.160** | **0.006** | **0.132** | **0.050** | -0.112 | 0.093 | 0.061 | 0.296 | 0.100 | 0.137 |
| Language | **0.244** | **0.000^*^** | **0.149** | **0.027** | **-0.193** | **0.004** | 0.005 | 0.938 | 0.119 | 0.076 |
| Abstraction | **0.289** | **0.000****^*^** | **0.132** | **0.049** | **-0.229** | **0.001^*^** | 0.002 | 0.973 | **0.180** | **0.007** |
| Delayed Memory | **0.151** | **0.009** | **0.206** | **0.002^*^** | -0.120 | 0.072 | 0.063 | 0.275 | 0.117 | 0.081 |
| Orientation | 0.080 | 0.171 | 0.102 | 0.131 | -0.087 | 0.190 | -0.061 | 0.297 | 0.009 | 0.893 |
| Other Non-Motor Symptoms |  |  |  |  |  |  |  |  |  |  |
| UPDRS-Ⅰ | -0.087 | 0.122 | -0.089 | 0.171 | -0.021 | 0.745 | **-0.349** | **0.000^*^** | **-0.224** | **0.000^*^** |
| HAMD | **-0.138** | **0.013** | **-0.137** | **0.034** | 0.051 | 0.430 | **-0.278** | **0.000^*^** | **-0.244** | **0.000^*^** |
| HAMA | **-0.151** | **0.007** | -0.102 | 0.117 | 0.063 | 0.326 | **-0.317** | **0.000^*^** | **-0.273** | **0.000^*^** |
| SCOPA-AUT | -0.029 | 0.605 | -0.106 | 0.103 | -0.087 | 0.174 | **-0.225** | **0.000^*^** | -0.102 | 0.113 |
| NMSS | **-0.149** | **0.008** | **-0.140** | **0.031** | 0.054 | 0.400 | **-0.346** | **0.000^*^** | **-0.287** | **0.000^*^** |
| AS | -0.021 | 0.762 | -0.102 | 0.201 | 0.058 | 0.459 | -0.043 | 0.527 | -0.091 | 0.244 |
| FSS | 0.080 | 0.355 | 0.054 | 0.548 | 0.011 | 0.896 | -0.091 | 0.286 | -0.112 | 0.204 |

**^*^** The *p*-value was survived after Bonferroni correction (*p*<0.002).

*Abbreviations*: *AS*, Apathy Scale; *FOG-Q*, Freezing of Gait Questionnaire; *FSS*, Fatigue Severity Scale; *HAMA*, Hamilton Anxiety Rating Scale; *HAMD*, Hamilton Depression Rating Scale; *H&Y,* Hoehn-Yahr stage; *LEDD*, levodopa equivalent daily dose; *UPDRS*, Movement Disorder Society Unified Parkinson’s Disease Rating Scale; *MMSE*, Mini-Mental State Examination; *MoCA*, Montreal Cognitive Assessment; *NMSS*, Non-motor symptoms Scales; *NSD,* nocturnal sleep duration; *PDQ-39*, 39-item Parkinson’s Disease Questionnaire; *PSQI*, Pittsburgh Sleep Quality Index; *SCOPA-AUT*, Scale for Outcomes in PD for Autonomic Symptoms; *TIB*, Total Time Spent in Bed.

**Supplementary Table 3. Effect estimates of total PSQI scores on PDQ-39 scores mediated via non-motor symptoms.**

| Characteristic | Effect Size | Indirect effect | p-value | Direct effect | p-value | Total effect | p-value | Proportion of total effect mediated | p-value | Ratio of indirect to direct effect |
| --- | --- | --- | --- | --- | --- | --- | --- | --- | --- | --- |
| MDS-UPDRS-I | β | 0.492 | **<0.001^*^** | 0.061 | 0.480 | 0.553 | **<0.001** | 0.865 | **<0.001^*^** | 8.124 |
|  | 95%CI | 0.39-0.62 |  | -0.12-0.19 |  | 0.34-0.74 |  | 0.69-1.32 |  |  |
| MMSE | β | 0.041 | **0.040** | 0.494 | **<0.001** | 0.535 | **<0.001** | 0.068 | **0.040** | 0.083 |
|  | 95%CI | 0.01-0.09 |  | 0.37-0.62 |  | 0.41-0.67 |  | 0.01-0.17 |  |  |
| MOCA | β | 0.019 | 0.160 | 0.518 | **<0.001** | 0.537 | **<0.001** | 0.031 | 0.160 | 0.037 |
|  | 95%CI | -0.01-0.05 |  | 0.35-0.66 |  | 0.36-0.68 |  | -0.01-0.09 |  |  |
| HAMD | β | 0.363 | **<0.001** | 0.184 | **<0.001** | 0.547 | **<0.001** | 0.670 | **<0.001^*^** | 1.976 |
|  | 95%CI | 0.27-0.49 |  | 0.06-0.33 |  | 0.40-0.68 |  | 0.46-0.86 |  |  |
| HAMA | β | 0.531 | **<0.001** | 0.019 | 0.600 | 0.550 | **<0.001** | 0.954 | **<0.001^*^** | 28.079 |
|  | 95%CI | 0.46-0.64 |  | -0.12-0.14 |  | 0.42-0.67 |  | 0.78-1.29 |  |  |
| SCOPA-AUT | β | 0.248 | **<0.001** | 0.325 | **<0.001** | 0.574 | **<0.001** | 0.429 | **<0.001^*^** | 0.763 |
|  | 95%CI | 0.18-0.35 |  | 0.18-0.48 |  | 0.41-0.73 |  | 0.30-0.58 |  |  |
| NMSS | β | 0.469 | **<0.001** | 0.061 | 0.320 | 0.530 | **<0.001** | 0.871 | **<0.001^*^** | 7.634 |
|  | 95%CI | 0.37-0.61 |  | -0.05-0.17 |  | 0.40-0.62 |  | 0.72-1.12 |  |  |
| AS | β | 0.063 | **<0.001** | 0.505 | **<0.001** | 0.568 | **<0.001** | 0.103 | **<0.001^*^** | 0.125 |
|  | 95%CI | 0.02-0.12 |  | 0.32-0.68 |  | 0.42-0.73 |  | 0.04-0.28 |  |  |
| FSS | β | 0.077 | **0.040** | 0.509 | **<0.001** | 0.587 | **<0.001** | 0.118 | **0.040** | 0.152 |
|  | 95%CI | 0.02-0.15 |  | 0.35-0.69 |  | 0.45-0.77 |  | 0.04-0.26 |  |  |

**^*^** The *p*-value was survived after Bonferroni correction (*p*<0.001).

*Abbreviations*: *AS*, Apathy Scale; *FSS*, Fatigue Severity Scale; *HAMA*, Hamilton Anxiety Rating Scale; *HAMD*, Hamilton Depression Rating Scale; *UPDRS*, Movement Disorder Society Unified Parkinson’s Disease Rating Scale; *MMSE*, Mini-Mental State Examination; n, number; *MoCA*, Montreal Cognitive Assessment; *NMSS*, Non-motor symptoms Scales; *PDQ-39*, 39-item Parkinson’s Disease Questionnaire; *PSQI*, Pittsburgh Sleep Quality Index; *SCOPA-AUT*, Scale for Outcomes in PD for Autonomic Symptoms.

**Supplementary Table 4. Effect estimates of total PSQI scores on H&Y stages mediated via non-motor symptoms.**

| Characteristic | Effect Size | Indirect effect | p-value | Direct effect | p-value | Total effect | p-value | Proportion of total effect mediated | p-value | Ratio of indirect to direct effect |
| --- | --- | --- | --- | --- | --- | --- | --- | --- | --- | --- |
| MDS-UPDRS-I | β | 0.309 | **<0.001** | 0.203 | 0.080 | 0.512 | **<0.001** | 0.558 | **<0.001^*^** | 1.522 |
|  | 95%CI | 0.18-0.46 |  | -0.05-0.41 |  | 0.29-0.71 |  | 0.36-1.23 |  |  |
| MMSE | β | 0.039 | **0.040** | 0.454 | **<0.001** | 0.493 | **<0.001** | 0.084 | **0.040** | 0.085 |
|  | 95%CI | 0.01-0.08 |  | 0.21-0.60 |  | 0.27-0.68 |  | 0.01-0.19 |  |  |
| MOCA | β | 0.016 | 0.280 | 0.463 | **<0.001** | 0.479 | **<0.001** | 0.025 | 0.280 | 0.034 |
|  | 95%CI | -0.01-0.06 |  | 0.25-0.70 |  | 0.26-0.71 |  | -0.02-0.13 |  |  |
| HAMD | β | 0.158 | **<0.001** | 0.372 | **<0.001** | 0.530 | **<0.001** | 0.293 | **<0.001^*^** | 0.424 |
|  | 95%CI | 0.08-0.26 |  | 0.16-0.61 |  | 0.31-0.76 |  | 0.14-0.58 |  |  |
| HAMA | β | 0.294 | **<0.001** | 0.208 | 0.080 | 0.502 | **<0.001** | 0.581 | **<0.001^*^** | 1.416 |
|  | 95%CI | 0.13-0.44 |  | -0.01-0.46 |  | 0.26-0.69 |  | 0.28-1.03 |  |  |
| SCOPA-AUT | β | 0.216 | **<0.001** | 0.283 | **0.040** | 0.499 | **<0.001** | 0.422 | **<0.001^*^** | 0.761 |
|  | 95%CI | 0.12-0.34 |  | 0.07-0.54 |  | 0.22-0.72 |  | 0.21-0.80 |  |  |
| NMSS | β | 0.402 | **<0.001** | 0.089 | 0.600 | 0.491 | **<0.001** | 0.826 | **<0.001^*^** | 4.521 |
|  | 95%CI | 0.27-0.57 |  | -0.18-0.34 |  | 0.26-0.68 |  | 0.43-1.61 |  |  |
| AS | β | 0.013 | 0.680 | 0.557 | **<0.001** | 0.570 | **<0.001** | 0.021 | 0.680 | 0.023 |
|  | 95%CI | -0.03-0.08 |  | 0.33-0.86 |  | 0.33-0.85 |  | -0.08-0.13 |  |  |
| FSS | β | 0.048 | **<0.001** | 0.091 | **0.040** | 0.139 | **<0.001** | 0.337 | **0.040** | 0.520 |
|  | 95%CI | 0.02-0.09 |  | 0.01-0.19 |  | 0.04-0.24 |  | 0.16-0.83 |  |  |

**^*^** The *p*-value was survived after Bonferroni correction (*p*<0.001).

*Abbreviations*: *AS*, Apathy Scale; *FSS*, Fatigue Severity Scale; *HAMA*, Hamilton Anxiety Rating Scale; *HAMD*, Hamilton Depression Rating Scale; *H&Y*, Hoehn-Yahr; *UPDRS*, Movement Disorder Society Unified Parkinson’s Disease Rating Scale; *MMSE*, Mini-Mental State Examination; n, number; *MoCA*, Montreal Cognitive Assessment; *NMSS*, Non-motor symptoms Scales; *PSQI*, Pittsburgh Sleep Quality Index; *SCOPA-AUT*, Scale for Outcomes in PD for Autonomic Symptoms.

**Supplementary Table 5. Effect estimates of total PSQI scores on UPDRS-Ⅱ scores mediated via non-motor symptoms.**

| Characteristic | Effect Size | Indirect effect | p-value | Direct effect | p-value | Total effect | p-value | Proportion of total effect mediated | p-value | Ratio of indirect to direct effect |
| --- | --- | --- | --- | --- | --- | --- | --- | --- | --- | --- |
| MDS-UPDRS-I | β | 0.383 | **<0.001** | 0.073 | 0.400 | 0.457 | **<0.001** | 0.858 | **<0.001^*^** | 5.222 |
|  | 95%CI | 0.28-0.49 |  | -0.08-0.22 |  | 0.32-0.64 |  | 0.62-1.28 |  |  |
| MMSE | β | 0.052 | **<0.001** | 0.426 | **<0.001** | 0.478 | **<0.001** | 0.094 | **<0.001^*^** | 0.121 |
|  | 95%CI | 0.01-0.10 |  | 0.30-0.54 |  | 0.33-0.62 |  | 0.02-0.19 |  |  |
| MOCA | β | 0.021 | 0.240 | 0.386 | **<0.001** | 0.407 | **<0.001** | 0.047 | 0.240 | 0.055 |
|  | 95%CI | -0.02-0.05 |  | 0.24-0.58 |  | 0.26-0.59 |  | -0.05-0.15 |  |  |
| HAMD | β | 0.174 | **<0.001** | 0.300 | **<0.001** | 0.474 | **<0.001** | 0.350 | **<0.001^*^** | 0.581 |
|  | 95%CI | 0.09-0.27 |  | 0.09-0.45 |  | 0.27-0.63 |  | 0.19-0.66 |  |  |
| HAMA | β | 0.314 | **<0.001** | 0.152 | 0.160 | 0.466 | **<0.001** | 0.661 | **<0.001^*^** | 2.066 |
|  | 95%CI | 0.23-0.44 |  | -0.02-0.35 |  | 0.33-0.63 |  | 0.44-1.05 |  |  |
| SCOPA-AUT | β | 0.283 | **<0.001** | 0.185 | **0.040** | 0.468 | **<0.001** | 0.600 | **<0.001^*^** | 1.535 |
|  | 95%CI | 0.18-0.38 |  | 0.03-0.38 |  | 0.29-0.64 |  | 0.39-0.92 |  |  |
| NMSS | β | 0.351 | **<0.001** | 0.071 | 0.360 | 0.422 | **<0.001** | 0.831 | **<0.001^*^** | 4.977 |
|  | 95%CI | 0.26-0.47 |  | -0.11-0.25 |  | 0.27-0.61 |  | 0.57-1.39 |  |  |
| AS | β | 0.034 | 0.080 | 0.481 | **<0.001** | 0.515 | **<0.001** | 0.074 | 0.080 | 0.071 |
|  | 95%CI | -0.01-0.07 |  | 0.32-0.64 |  | 0.35-0.65 |  | -0.02-0.15 |  |  |
| FSS | β | 0.061 | **<0.001** | 0.345 | **<0.001** | 0.405 | **<0.001** | 0.128 | **<0.001^*^** | 0.176 |
|  | 95%CI | 0.01-0.16 |  | 0.11-0.58 |  | 0.23-0.64 |  | 0.03-0.39 |  |  |

**^*^** The *p*-value was survived after Bonferroni correction (*p*<0.001).

*Abbreviations*: *AS*, Apathy Scale; *FSS*, Fatigue Severity Scale; *HAMA*, Hamilton Anxiety Rating Scale; *HAMD*, Hamilton Depression Rating Scale; *UPDRS*, Movement Disorder Society Unified Parkinson’s Disease Rating Scale; *MMSE*, Mini-Mental State Examination; n, number; *MoCA*, Montreal Cognitive Assessment; *NMSS*, Non-motor symptoms Scales; *PSQI*, Pittsburgh Sleep Quality Index; *SCOPA-AUT*, Scale for Outcomes in PD for Autonomic Symptoms.

**Supplementary Table 6. Effect estimates of total PSQI scores on UPDRS-Ⅲ scores mediated via non-motor symptoms.**

| Characteristic | Effect Size | Indirect effect | p-value | Direct effect | p-value | Total effect | p-value | Proportion of total effect mediated | p-value | Ratio of indirect to direct effect |
| --- | --- | --- | --- | --- | --- | --- | --- | --- | --- | --- |
| UPDRS-I | β | 0.147 | **<0.001** | 0.075 | 0.360 | 0.221 | **<0.001** | 0.680 | **<0.001^*^** | 1.964 |
|  | 95%CI | 0.06-0.22 |  | -0.07-0.24 |  | 0.10-0.35 |  | 0.22-1.65 |  |  |
| MMSE | β | 0.044 | **<0.001** | 0.136 | 0.160 | 0.478 | **<0.001** | 0.244 | **<0.001^*^** | 0.326 |
|  | 95%CI | 0.01-0.07 |  | -0.01-0.26 |  | 0.03-0.31 |  | 0.071.36 |  |  |
| MOCA | β | 0.022 | 0.120 | 0.134 | 0.120 | 0.157 | 0.040 | 0.110 | 0.160 | 0.164 |
|  | 95%CI | -0.01-0.06 |  | -0.02-0.30 |  | 0.01-0.33 |  | -0.13-1.43 |  |  |
| HAMD | β | 0.082 | **<0.001** | 0.119 | 0.120 | 0.201 | **<0.001** | 0.400 | **<0.001^*^** | 0.688 |
|  | 95%CI | 0.03-0.17 |  | -0.04-0.27 |  | 0.07-0.32 |  | 0.11-1.29 |  |  |
| HAMA | β | 0.167 | **<0.001** | 0.018 | 0.840 | 0.185 | **<0.001** | 0.905 | **<0.001^*^** | 9.210 |
|  | 95%CI | 0.07-0.29 |  | -0.12-0.19 |  | 0.07-0.31 |  | 0.31-2.58 |  |  |
| SCOPA-AUT | β | 0.115 | **<0.001** | 0.085 | 0.200 | 0.200 | **<0.001** | 0.574 | **<0.001^*^** | 1.349 |
|  | 95%CI | 0.05-0.18 |  | -0.03-0.18 |  | 0.08-0.31 |  | 0.23-1.36 |  |  |
| NMSS | β | 0.158 | **<0.001** | 0.028 | 0.640 | 0.186 | **<0.001** | 0.844 | **<0.001^*^** | 5.585 |
|  | 95%CI | 0.10-0.22 |  | -0.08-0.13 |  | 0.09-0.27 |  | 0.53-1.66 |  |  |
| AS | β | 0.024 | 0.080 | 0.220 | **0.040** | 0.244 | **0.040** | 0.079 | 0.120 | 0.108 |
|  | 95%CI | -0.01-0.06 |  | 0.04-0.36 |  | 0.06-0.38 |  | -0.02-0.33 |  |  |
| FSS | β | 0.042 | **0.040** | 0.198 | 0.080 | 0.240 | **0.040** | 0.151 | 0.080 | 0.211 |
|  | 95%CI | 0.01-0.10 |  | -0.01-0.36 |  | 0.06-0.40 |  | -0.04-0.61 |  |  |

**^*^** The *p*-value was survived after Bonferroni correction (*p*<0.001).

*Abbreviations*: *AS*, Apathy Scale; *FSS*, Fatigue Severity Scale; *HAMA*, Hamilton Anxiety Rating Scale; *HAMD*, Hamilton Depression Rating Scale; *UPDRS*, Movement Disorder Society Unified Parkinson’s Disease Rating Scale; *MMSE*, Mini-Mental State Examination; n, number; *MoCA*, Montreal Cognitive Assessment; *NMSS*, Non-motor symptoms Scales; *PSQI*, Pittsburgh Sleep Quality Index; *SCOPA-AUT*, Scale for Outcomes in PD for Autonomic Symptoms.
